# Supplementary figures and images for: Integral methods for automatic quantification of fast-scan-cyclic-voltammetry detected neurotransmitters
Source: PLoS One. 2021 Jul 26;16(7):e0254594. doi: 10.1371/journal.pone.0254594 (PMC8312965; doi:10.1371/journal.pone.0254594)

(a)
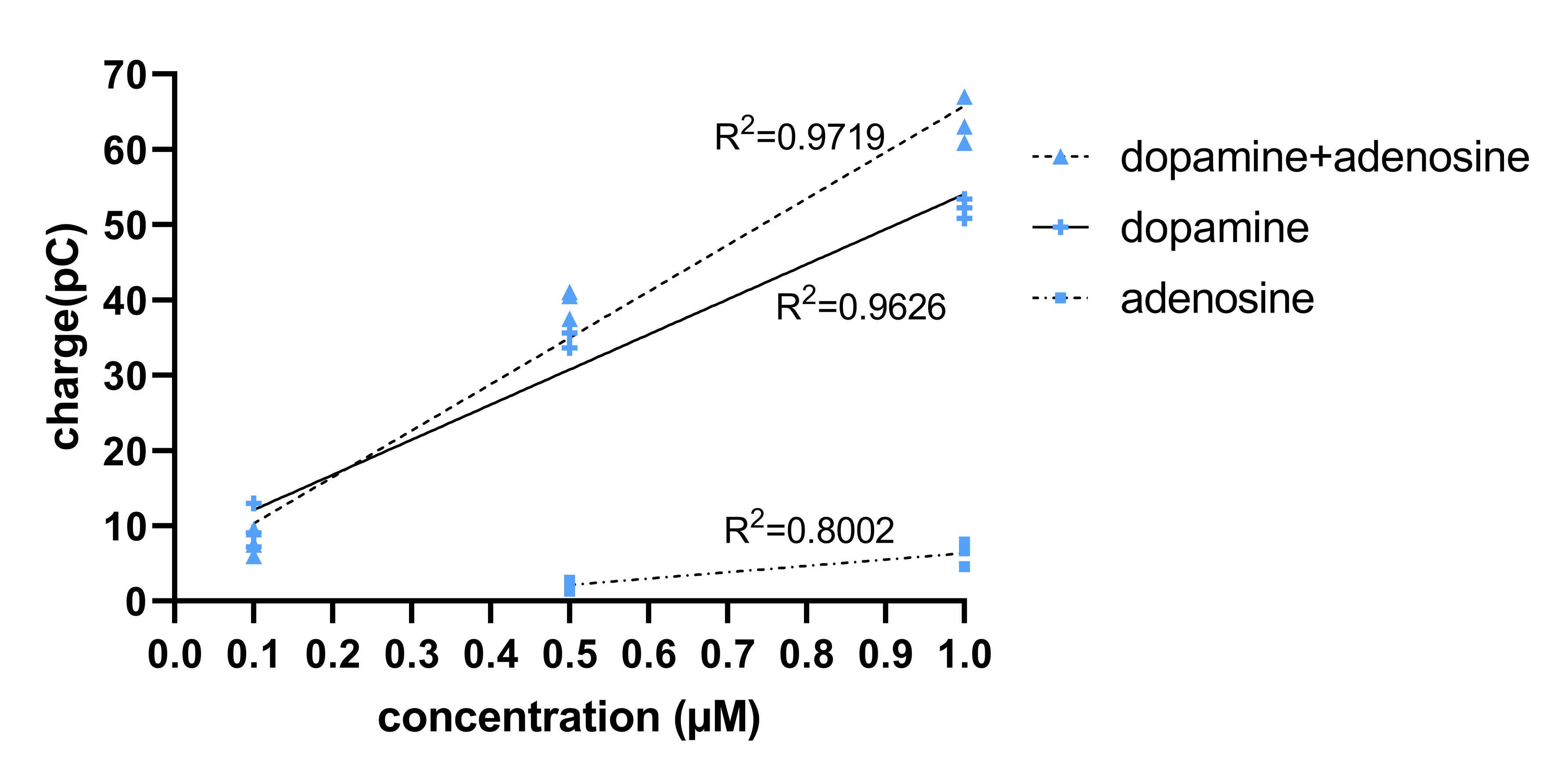


(b)
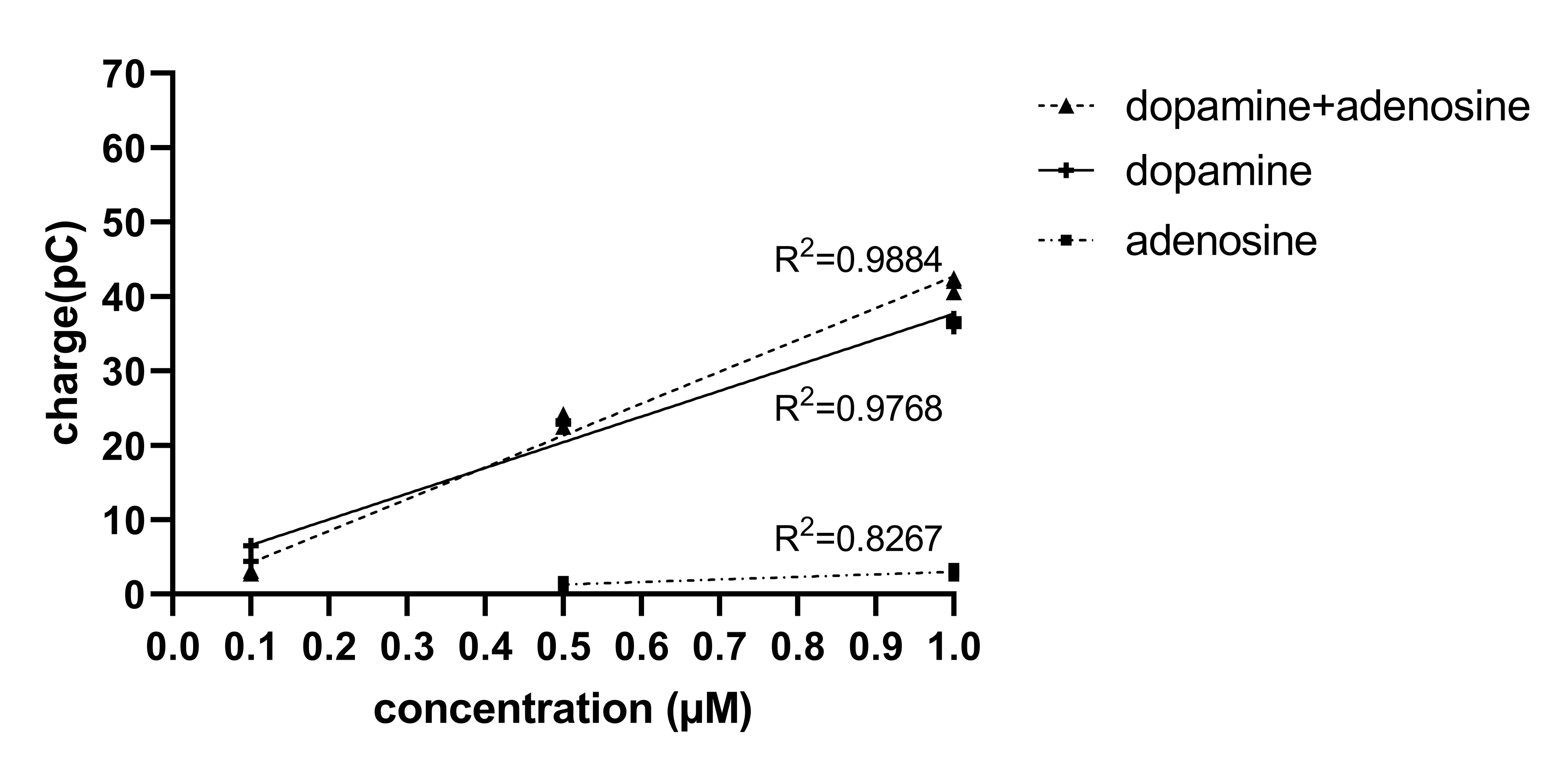


(c)
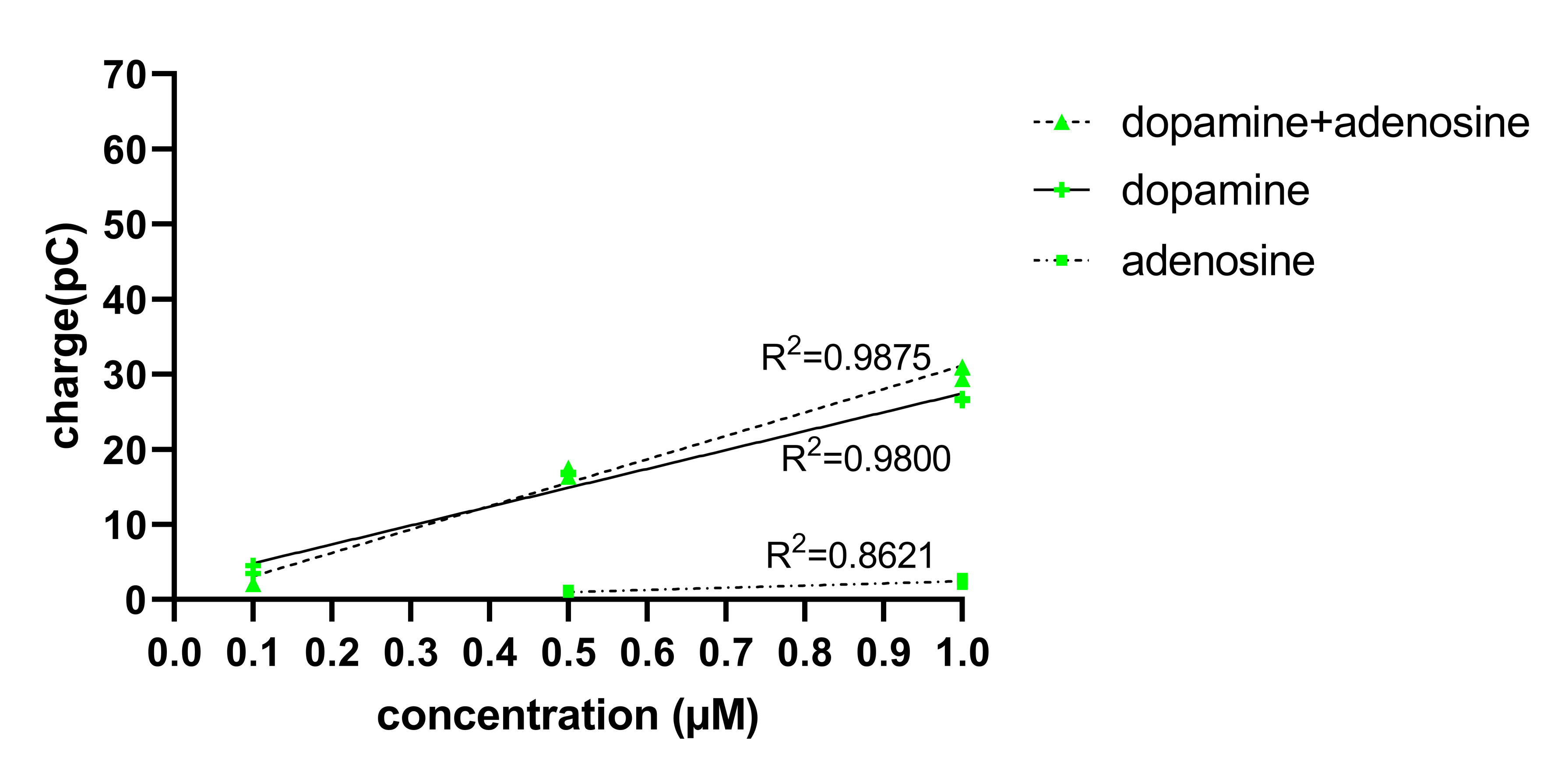

Supplement: S1 Fig — Data are fit with a linear regression and R2 values are displayed for each analyte. (DOCX) [file pone.0254594.s001.docx]
