# Supplementary material for: Integral methods for automatic quantification of fast-scan-cyclic-voltammetry detected neurotransmitters
Source: PLoS One. 2021 Jul 26;16(7):e0254594. doi: 10.1371/journal.pone.0254594 (PMC8312965; doi:10.1371/journal.pone.0254594)
Supplement: Attachment — Submitted filename: Response to reviewers.docx [file pone.0254594.s002.docx]

5. Review Comments to the Author

Please use the space provided to explain your answers to the questions above. You may also include additional comments for the author, including concerns about dual publication, research ethics, or publication ethics. (Please upload your review as an attachment if it exceeds 20,000 characters)

Reviewer #1: This study discusses the development of a novel methodology for selecting integration limits for the purpose of quantifying the concentration of electrochemical targets using FSCV. This technology advancement could serve as a critical improvement to the in vivo basal detection method FSCAV by replacing user-defined integration limits with algorithm-defined cutoffs. This would have the desired effect of removing the possibility for user bias from FSCAV data analysis. This study is highly relevant but requires substantial revision prior to publication.

Major

• How does the curvature method perform when dealing with small in vivo oxidation peaks resulting from FSCAV basal detection or brief DBS stimulation? The authors state that the desired use for is for this methodology is to provide cutoff limits for in vivo FSCAV experiments. This study should show the applicability of the methodology using FSCAV data.

The goal was to identify integration limits for charge calculations of FSCV data. While a comparison of integration limits between FSCV and FSCAV would be interesting, analysis of FSCAV data is outside the scope of the study.

• The responses in Figure 3 display varying degrees of linearity (none of them highly linear). Please discuss the consequences of this given that linear regression of these responses is used to convert between charge and concentration for unknown samples. Does the degree of linearity for the various methods adjust the determination of which method is best suited for use?
We can include this point in the “challenges and limitations” paragraph suggested by reviewer two.

The following was included in the “limitations and challenges” paragraph. “The relationship between charge and analyte concentration is nonlinear (Figure 3). While linear regressions are regularly used when creating a calibration curves [6, 7, 8, 9, 10], this approach may result in underestimations at the low and high ends of detectable concentrations while overestimations concentrations at the midpoint of the detectible range. Fitting with a nonlinear regression model may reduce these errors when converting between charge and concentration for unknown samples.”
• Please provide detailed statistical analysis between groups to support claims. For example, the author’s claim that there is a “significant difference between average left curvature boundaries for DA and NEP” is not supported by statistical evidence.

Discuss with Luis. We thank the reviewer for pointing out…The text has been changed to the following: “Indeed, panels (b) and (c) of figure 2 indicate that there is a significant difference between average left curvature boundaries for the 1 µM dopamine and 1 µM norepinephrine voltammograms as demonstrated using an unpaired two-tailed t-test with welch’s correction (p=.0002)”.

• There are many cases where the authors neglect discussing data sets within the figures. For example, the authors largely neglect discussing the data arising from the inflection method throughout the entire manuscript as well as the FSCAV limits for EPI. Please discuss all relevant data provided in the figures.

We thank the reviewer for pointing out these omissions. Relevant data provided in figures is discussed in the manuscript, including data arising from the inflection method. FSCAV limits for Epinephrine are shown in figure 4b.

• The authors state that “A region of positive, nearly constant current between 1 and 1.5 volts that persists far into the cathodic sweep is likely the consequence of background drift.” Is there evidence to support that claim? If the feature is drift, why does that drift not occur prior to the oxidation peak (-0.4 V to 0.3 V)?

We thank the reviewer for pointing this underexplained feature of figure 5. The following statement has been added to support this claim. “Drift is exacerbated by disruption of Helmholtz layer or pH changes as a result of electrical stimulation. The integration boundaries can still be calculated in the presence of background drift, though the exact cutoffs may be affected. It is generally advisable for the experimenter to minimize any possible sources of background drift or other redox sources in FSCV recordings for consistent data analysis and interpretation.”

• What is meant by the “random variation of the DBS stimulation parameters” in the main text when describing the data in Figure 5. Were these data collected using the various duty cycles detailed in Figure 6? What set of conditions were used to produce the response in Figure 5a?

We thank the reviewer for pointing out our omission of DBS stimulation parameters in the manuscript…we have clarified in the class to indicate in the text that… “Stimulation amplitude and pulse widths ranging from 0.14-0.16ms and 1.37-1.57ms, respectively, were systematically applied in a random order to minimize effects of hysteresis. All stimulation epochs consisted of 180 pulses delivered at 90Hz”. The following description of stimulation parameters was added to figure 6. “Stimulation parameters for the traces X,Y, and Z are as follows X: 0.14mA, 1.46ms pulse width, 180 pulses, 90Hz Y: 0.16mA, 1.57ms pulse width, 180 pulses, 90Hz Z: 0.15mA, 1.45ms pulse width, 180 pulses, 90Hz” All parameters are shown below in sequential order of delivery will be included in the data repository:

| Stimulation number | phase 1 amplitude (mA) | phase 1 duration (milliseconds) | phase 1 interval (milliseconds) | phase 2 amplitude (mA) | phase 2 duration (milliseconds) | phase 2 interval (milliseconds) |
| --- | --- | --- | --- | --- | --- | --- |
| 1 | 0.14 | 1 | 0.025 | 0.14 | 1 | 8.2214 |
| 2 | 0.14 | 1 | 0.025 | 0.14 | 1 | 8.1731 |
| 3 | 0.14 | 1 | 0.025 | 0.14 | 1 | 8.1387 |
| 4 | 0.15 | 1 | 0.025 | 0.15 | 1 | 8.0736 |
| 5 | 0.14 | 1 | 0.025 | 0.14 | 1 | 8.1347 |
| 6 | 0.15 | 1 | 0.025 | 0.15 | 1 | 8.1167 |
| 7 | 0.14 | 1 | 0.025 | 0.14 | 1 | 8.1595 |
| 8 | 0.14 | 1 | 0.025 | 0.14 | 1 | 8.161 |
| 9 | 0.14 | 1 | 0.025 | 0.14 | 1 | 8.1738 |
| 10 | 0.14 | 1 | 0.025 | 0.14 | 1 | 8.1582 |
| 11 | 0.14 | 1 | 0.025 | 0.14 | 1 | 8.1469 |
| 12 | 0.15 | 1 | 0.025 | 0.15 | 1 | 8.1272 |
| 13 | 0.14 | 1 | 0.025 | 0.14 | 1 | 8.1826 |
| 14 | 0.14 | 1 | 0.025 | 0.14 | 1 | 8.1503 |
| 15 | 0.14 | 1 | 0.025 | 0.14 | 1 | 8.1544 |
| 16 | 0.15 | 1 | 0.025 | 0.15 | 1 | 8.1229 |
| 17 | 0.14 | 1 | 0.025 | 0.14 | 1 | 8.1747 |
| 18 | 0.14 | 1 | 0.025 | 0.14 | 1 | 8.1432 |
| 19 | 0.15 | 1 | 0.025 | 0.15 | 1 | 8.1093 |
| 20 | 0.14 | 1 | 0.025 | 0.14 | 1 | 8.1984 |
| 21 | 0.15 | 1 | 0.025 | 0.15 | 1 | 8.0965 |
| 22 | 0.16 | 1 | 0.025 | 0.16 | 1 | 7.9723 |
| 23 | 0.16 | 1 | 0.025 | 0.16 | 1 | 7.9533 |
| 24 | 0.15 | 1 | 0.025 | 0.15 | 1 | 8.0753 |
| 25 | 0.15 | 1 | 0.025 | 0.15 | 1 | 8.0628 |
| 26 | 0.16 | 1 | 0.025 | 0.16 | 1 | 8.0119 |
| 27 | 0.16 | 1 | 0.025 | 0.16 | 1 | 8.0162 |
| 28 | 0.15 | 1 | 0.025 | 0.15 | 1 | 8.1602 |
| 29 | 0.15 | 1 | 0.025 | 0.15 | 1 | 8.2072 |
| 30 | 0.15 | 1 | 0.025 | 0.15 | 1 | 8.1774 |
| 31 | 0.15 | 1 | 0.025 | 0.15 | 1 | 8.2222 |
| 32 | 0.14 | 1 | 0.025 | 0.14 | 1 | 8.312 |
| 33 | 0.14 | 1 | 0.025 | 0.14 | 1 | 8.2973 |
| 34 | 0.15 | 1 | 0.025 | 0.15 | 1 | 8.2457 |
| 35 | 0.15 | 1 | 0.025 | 0.15 | 1 | 8.2641 |
| 36 | 0.14 | 1 | 0.025 | 0.14 | 1 | 8.3466 |
| 37 | 0.15 | 1 | 0.025 | 0.15 | 1 | 8.3025 |
| 38 | 0.15 | 1 | 0.025 | 0.15 | 1 | 8.2837 |
| 39 | 0.15 | 1 | 0.025 | 0.15 | 1 | 8.2858 |
| 40 | 0.15 | 1 | 0.025 | 0.15 | 1 | 8.3141 |
| 41 | 0.15 | 1 | 0.025 | 0.15 | 1 | 8.3175 |

• Are the results detailed in Figure 6b meant to state that the increase in the charge is due to an increase in the extracellular concentration of the in vivo chemical (I assume the chemical is dopamine but the authors never state the identity of the in vivo signal) or is this a consequence of the duty cycle artifact on integration limits? In addition, the authors neglect discussing how the data seem to group by stimulation current as well. Please substantially revise and clarify this discussion.

The *in vivo* signal is likely dopamine based on the REDOX peaks and the simulating electrode location in the dopaminergic afferents of the medial forebrain bundle and CFM recording location in the striatum. This signal identity is now explicitly stated in figure 6 and in the text.

• It is unclear why the authors would want to optimize quantifying stimulation artifacts in Figure 6. Stimulation timing is routinely staggered with FSCV recordings to ensure that artifacts do not distort background subtracted voltammograms. Please explain.

We agree with the reviewer’s note about the common practice of interleaving DBS waveforms with FSCV acquisition such that DBS artifacts are minimized. With this in mind, we adjusted the text body to state that figure 6 is “…,demonstrating that the integration limits can be objectively determined with varying stimulation parameters, even in the presence of sources of noise such as a stimulation artifact.”

• The 2nd EPI peak in Figure 8b has clearly not resolved by the switching potential. Cutting at the switching potential does not seem appropriate for obtaining an accurate quantification of the 2nd peak. Is this technique only applicable for targets where the peaks are allowed to totally resolve prior to the switching potential?

You are correct in point out that the peak at the reversal potential in figure 8b may not be resolved. However, epinephrine is a secondary amine with a second oxidation peak for adenosine is near the potential at which water oxidizes, making it difficult to resolve (Venton and Cao 2020; Chan et al. 2020). This technique is appropriate for neurochemicals with at least one resolvable peak.

• How does the sensitivity for target (DA) detection in the presence of interferent compare to the sensitivity for target alone using the various methods? This is critical to assessing the selectivity of the technique. Please provide the linear calibration data for DA in the presence of interferent (Figure 8a) and compare to response with DA alone.

The reviewers bring up an important point here. We have included a supplementary figure showing the linear calibration data for oxidation charges calculated using “true”, “inflection”, and “curvature” limits of Dopamine, Adenosine, and Dopanine+Adenosine at 0.1 µM, 0.5µM, and 1µM. Adenosine was not resolvable at 0.1µM, and as such, was not plotted.

Supplemental Figure 1: Linear charge calibration curve for dopamine, adenosine, and dopamine +adenosine using a)True integration limits b) curvature integration limits, and c) inflection integration limits at concentrations of 0.1 µM, 0.5 µM, and 1 µM. Data are fit with a linear regression and R^2^ values are displayed for each analyte.

(a)

(b)

(c)

Minor

• Please state the specific “computational routines for filtering and smoothing” used in this study.
A 1000Hz lowpass 2^nd^ phase butterworth filter was applied to all data in both forward and reverse directions to reduce spurious signals. Data was also smoothed with a 5-point moving average and a CSAPS Cubic smoothing spline before background subtraction subsequent charge calculations.

• How many individual electrodes were tested for both in vitro and in vivo testing? How many animals were used for in vivo testing?

The in vivo data shown was collected from one animal with one CFM. The in vitro data were collected from two electrodes. This is now indicated in the text.

• How is “accuracy” determined when comparing calculated charges from FSCAV limits and curvature limits to the “True Charges”? Does this refer to linear sensitivity? Please be more specific.

Use of the word “accuracy” has been replaced with specific statistical statements about the data.

• It is notable that the DA, NEP and EPI experiments in Figures 2, 3 and 4 reflect different experimental designs. Why was NEP and EPI tested up to 5 µM whereas DA was only tested to 1 µM? Why were there so few replicates for NEP and EPI as compared to DA? If the authors intend to provide a fair comparison between the responses of various chemicals then the experimental protocols should mimic each other.

Reviewer 1 is correct in pointing out that experimental designs of figures 2,3, and 4 vary. The reason it is not critical in this case is because as a lab we are interested in developing this approach in the context of Dopamine, which is often implicated in Parkinson’s disease and other neurological conditions. Consequently, we have larger datasets of DA than other electroactive chemicals. NEP, EPI, and Adenosine were selected as possible interferents that would interfere with Dopamine. NEP and EPI have lower sensitivities than Dopamine with FSCV, and as such, higher concentrations were explored. `

• The authors state that “Figure 4 shows an example were curvature integration boundaries provide the best results among the four methods considered”. I only see 3 methods represented (please include the “True” results. Also, what does “best results” mean?

The text has been changed to include the following quantitative description of figure 4 “Curvature integration boundaries provide the highest charge among the three methods considered at concentrations above 0.5 µM as shown by a comparison of curvature and FSCAV datapoints using an unpaired two-tailed t-test with welch’s correction (0.1µM, p>0.05; 0.5µM, p=.024; 1µM, p=.0001; 5 µM, p=.0003)(Figure 4).”

• Please show the peak shape for NEP since the peak shape is shown for DA and EPI.

In the interest of minimizing the length of this article, we have decided to omit a NEP voltammogram figure while directing the reader to references where Norepinephrine voltammograms are described in [18,19].

• Why are average values for the inflection method not displayed on Figures 3 and 4?

To reduce the amount of text within the figures 3 and 4, average and standard deviations were not plotted for the inflection method. Individual data points are, however, still included.

• Please provide markings for all four of the limits on Figure 4a (similar to Figure 1a)

The curvature method results in a closer approximation to true charge than the other methods. Consequently, all analyses of charge in subsequent figures are performed using the curvature method for purposes of focus and clarity.

• The a) and b) labeling on Figures 1 and 5 are not consistent with the other figures.

Thank you for pointing out this inconsistency. The labels of (a) and (b) on Figures 1 and 5 have been modified to be consistent with other figures.

• How do the in vivo limits compare to the previous limits defined by FSCAV? Please incorporate those cutoff values into Figures 5a and 5b.
As described previously in the response to the comment on Figure 4a, the curvature method results in a closer approximation to true charge than the other methods. Consequently, all analyses of charge in subsequent figures are performed using the curvature method for purposes of focus and clarity.

• Average ± What? Is it standard deviation? Standard error?

We have clarified ± represents standard deviation in all relevant figures.

• What do the numbers in the legend of Figure 6a stand for?
We have clarified in figure 6 that numbers represent time at which stimulation occurred.

• The authors state that there cannot be a “True Limit” for the reduction peak. They previously stated that the switching potential could serve as a limit for the “True” method in cases where the peak does not return to zero (i.e. in vivo data). Wouldn’t this justification be applicable here as well and allow for inclusion of the “True” data set into Figure 7?
In many cases, the reduction peak does not return to cross zero at the right end of the anodic sweep (e.g. right most data point of figure 1a) which prohibits the identification and inclusion of “True” data points for the reduction peaks in figure 7.

• It is confusing why the two background-subtracted voltammograms for EPI (Figure 4a and Figure 8b) look so different. Are they at different concentrations? Why is the ratio of the two oxidation peak heights so drastically different between the two responses?

Thank you for pointing out this difference in the epinephrine voltammograms in 4a and 8b. This difference is attributable to varying concentrations where 4a shows a voltammogram of 1 *µ*M epinephrine while 8b is of 0.5 *µ*M epinephrine. This has been clarified in the figure captions.

Reviewer #2: This paper presents a novel approach to identify neurochemicals and a clever effort to reduce false observations in FSCV or FSCAV data. Given that most of voltammetric post-analysis methods are prone to misinterpretation, a more mathematical approach – like the one presented in this work – can contribute to making more accurate statements on the collected biological data. It would be interesting to see how this work would develop in future.
The paper is well written and the title fits the premises of the target study. I have several rather technical comments to further improve the clarity of the presented method/results (listed here in the order of appearance).

Abstract-------------------------------

1. In the last paragraph, the authors claimed, “Our results show that these methods allow quantification of oxidation and reduction reactions, for multiple analytes, both in vitro and in vivo.” however:
a) the provided results in the manuscript do not fully address in vivo quantification of the reduction reactions (pages 10,3.3). Please correct the text or include more data to support the claim.

The text has been changed to “Our results show that these methods allow quantification of oxidation reactions both *in vitro* and *in vivo* and of multiple analytes *in vitro*”

b) the provided data in the manuscript do not support the claim on detection of “multiple analytes” in vivo (see page 10, section 3.2, Figure 6). Please amend the text to better reflect the result or include more data to support the text.

The text has been changed to “Our results show that these methods allow quantification of oxidation reactions both *in vitro* and *in vivo* and of multiple analytes *in vitro*”

Method--------------------------------

2. Please specify what is meant by “A 110 µm CFM”. Both the length and diameter of the used electrode should be provided (page 2, 2.1)
The text has been changed to “ A CFM of 7µm diameter and110µm in length”
3. The authors have presented this work as an automatic quantification approach however, the manuscript is missing details on how theses boundary conditions were implemented into an automatized system. Authors should support the concept with a schematic flowchart/algorithm of the applied “routines”. The flowchart should provide an overview of the applied computational procedure for filtering/smoothing and background- subtractions in addition to the correction routines (page 5, 2.3).

We have included the following schematic (figure 1c) to clarify the workflow:

Results and discussion-------------------------------

4. According to the manuscript, 70 injections of DA and 15 injections of norepinephrine were applied to produce the results (page 5, 3.1). However, it is not clear how many electrodes were used to generate the presented data. Please provide the sample size, i.e. number of electrodes in addition to the number of injections per electrode, for the presented results.

(c)

The *in vivo* data were each collected using one CFM. The *in vitro* data was collected using two electrodes, with 3-15 injections per analyte.

5. Statistical analysis (including the level of significance) should be included to support the presented comparisons. An optical judgment is indeed a good cue but not sufficient for deciding on the efficacy of the presented method. Here are some example statements which should be statistically backed up:
“In Figure 4 shows an example were curvature integration boundaries provide the best results among the four methods considered” (page 8, 3.1).

The text has been changed to include the following quantitative description of figure 4 “Curvature integration boundaries provide the highest charge among the three methods considered at concentrations above 0.5 µM as shown by a comparison of curvature and FSCAV datapoints using an unpaired two-tailed t-test with welch’s correction (0.1µM, p>0.05; 0.5µM, p=.024; 1µM, p=.0001; 5 µM, p=.0003)(Figure 4).”

“Figure 3(b) shows that oxidation charges obtained with curvature limits provide a better approximation to true charges than FSCAV limits.” (page 7, 3.1)

The text has been changed to include the following quantitative description of figure 3b “Oxidation charges obtained with curvature limits provide a closer approximation to true charges than FSCAV limits at concentrations of 1µM and greater, as demonstrated by a comparison of mean curvature and FSCAV datapoints using an ANOVA with Holm-Sidak comparison for multiple corrections (at 0.5µM, curvature (20.58±0.99) v. FSCAV(19.48±0.88), P=0.1087 n.s.; at 1µM, curvature (86.84±2.21) v. FSCAV(75.57±1.76), P<0.0001 ****; at 5µM, curvature (27.23±0.6) v. FSCAV(24.88±0.0.56), P<0.01 ****) (Figure 3b).”

“…curvature boundaries select a wider region than that enclosed by the injection boundaries (figure 1), and they provide a better approximation to the charges obtained by using true boundaries,..” (page 7, 3.1)

This sentence was not critical for the message of the manuscript and has been removed from the text body.

6. Please include one paragraph discussing the limitations and challenging aspects of the proposed approach.

The following section titled “3.5 Challenges and Limitations ” has been added:

“While this approach may be helpful for standardizing oxidation charge quantification of electroactive neurochemicals, it is not without limitations. The relationship between charge and analyte concentration is nonlinear (Figure 3). While linear regressions are regularly used when creating a calibration curves [6, 7, 8, 9, 10], this approach may result in underestimations at the low and high ends of detectable concentrations while overestimations concentrations at the midpoint of the detectible range. Fitting with a nonlinear regression model may reduce these errors when converting between charge and concentration for unknown samples. Furthermore, xxxx. Despite these limitations, computational approaches such as the one described here are necessary for improving accuracy and consistency of electroactive neurochemical measurements.”
